# Minimal prognostic significance of sentinel lymph node metastasis in patients with cT1–2 and cN0 breast cancer

**DOI:** 10.1186/s12957-019-1585-9

**Published:** 2019-02-23

**Authors:** Hideo Shigematsu, Mai Nishina, Daisuke Yasui, Taizo Hirata, Shinji Ozaki

**Affiliations:** 1 Department of Breast Surgery, National Hospital Organization Kure Medical Center and Chugoku Cancer Center, 3-1, Aoyama-cho, Kure City, Hiroshima 737-0023 Japan; 2 Department of Medical Oncology, National Hospital Organization Kure Medical Center and Chugoku Cancer Center, Kure City, Hiroshima Japan

**Keywords:** Breast cancer, SLN, Minimal, Prognosis, Proliferation marker

## Abstract

**Background:**

The prognostic value of sentinel lymph node (SLN) metastases may be minimized by the limited disease burden of lymph node metastases and tailoring adjuvant therapy based on breast cancer biology. The aim of this study is to assess the prognostic significance of SLN metastasis in patients with cT1–2N0M0 breast cancer.

**Patients and methods:**

Between January 2006 and December 2015, 582 patients underwent SLN biopsy for cT1–2N0M0 breast cancers. cN0 was essentially diagnosed by ultrasound sonography. The prognostic values of SLN metastases were retrospectively evaluated.

**Results:**

Among 582 patients with cT1–2N0M0 breast cancer, 111 patients (19.1%) were positive for SLN metastasis, including 39 cases (6.7%) of micrometastasis and 72 cases (12.4%) of macrometastases. The median size of SLN metastasis was 3.0 mm (range 0.2–16 mm, mean 4.1 mm). In log-rank test, presence of SLN metastasis was not associated with breast cancer recurrence (*p* = 0.21); 5-year and 10-year recurrence-free survival (RFS) were 93.0% and 96.5%, and 93.0% and 90.4% in the SLN-positive and SLN-negative groups, respectively. In the propensity score matching cohort (*n* = 178), there was no significant difference in RFS between the SLN-positive and SLN-negative groups (*p* = 0.90). In Cox regression analysis, a continuous value of Ki67 expression was a significant prognostic factor (HR 1.03; 95% CI 1.01–1.05, *p* = 0.017).

**Conclusion:**

SLN metastasis has a minimal impact on RFS for patients with cT1–2N0M0 breast cancer in the modern medical era. A proliferation marker is a better factor for poor prognosis than the presence of SLN metastases in this population.

**Electronic supplementary material:**

The online version of this article (10.1186/s12957-019-1585-9) contains supplementary material, which is available to authorized users.

## Introduction

Sentinel lymph node (SLN) biopsy is a standard procedure for assessing axillary lymph node metastasis in a patient with clinically node-negative (cN0) breast cancer [1, 2]. Although the therapeutic value of axillary surgery is denied in patients with clinical T1–T2N0 (cT1–2N0) breast cancer [2, 3], pathological nodal status is still an important poor prognostic factor for decisions regarding adjuvant therapy. In a patient with SLN metastasis, adjuvant chemotherapy and radiotherapy are administered in cases with clinically node-positive (cN+) breast cancer. However, the prognostic value of SLN metastasis in patients with cT1–2N0M0 breast cancer has become controversial with limited disease burden in SLN due to the advances in preoperative axillary evaluation and the introduction of modern tailored therapy based on breast biology [4]. A previous report showed that axillary ultrasound sonography (AUS) for patients with cT1–2 breast cancer had a sensitivity of 70% and a negative predictive value of 84% for detection of lymph node involvement [5]. Meta-analysis of AUS-guided needle cytology or biopsy of axillary lymph nodes in patients with invasive breast cancer estimated 79.6% of sensitivity, 98.3% of specificity, and 97.1% of positive predictive value for axillary staging [6]. AUS-guided cytology or biopsy of suspicious lymph node was also shown to accurately exclude clinically significant lymph node metastasis [7, 8]. Patients with cN0 breast cancer diagnosed by AUS are expected to have a limited disease burden of the axilla, which raises the hypothesis that the presence of SLN metastasis has the minimal prognostic value. Although the anatomical stage of breast cancer staging still has clinical significance, breast cancer biology has become the most important prognostic and predictive factor for determining adjuvant therapy [9]. Tailoring adjuvant therapy based on breast cancer biology has resulted in the significant improvement of prognosis in early-stage breast cancer [10, 11]. Considering advances in preoperative axillary evaluation and tailored therapy based on breast biology, the prognostic value of SLN metastasis in cN0 breast cancer should be reevaluated.

The aim of this study is to assess the prognostic value of SLN metastases in patients with cT1–2N0M0 breast cancer in the modern medical era. We retrospectively evaluated the prognostic impact of SLN metastases in this population.

## Materials and methods

### Patients and methods

A total of 582 consecutive patients with clinically T1–2N0M0 invasive breast cancers underwent sentinel lymph node biopsy (SLNB) for axillary staging at the Kure Medical Center and the Chugoku Cancer Center, Kure, Japan, between January 2006 and December 2015. This study retrospectively evaluated the prognostic value of SLN metastasis. The Kure Medical Center review board approved this study (30-05). The requirement for informed consent from individual patients was waived because this was a retrospective review of a prospectively maintained patient database.

### Clinicopathological factors

The clinicopathological factors in this retrospective study retrieved from our prospectively maintained database included age at surgery, T factor, SLN status, estrogen receptor (ER) status, progesterone receptor (PgR) status, human epidermal growth factor receptor 2 (HER2) status, nuclear grade, Ki67 index, and prescriptions of adjuvant chemotherapy/radiotherapy. ER and PgR status were evaluated by immunohistochemical (IHC) assays, and ≥ 1% positively stained tumor cells were classified as positive. HER2 status was evaluated by IHC and fluorescence in situ hybridization (FISH) individually or in combination. HER2-positive tumors were defined as those with an IHC score of 3+ or those showing HER2 gene amplification using FISH, in accordance with the ASCO guidelines [12].

Adjuvant systemic and/or radiation therapy was administered as clinically indicated [9]. Patients with hormone receptor (HR)-positive breast cancers were treated with adjuvant endocrine therapy for at least 5 years. Administration of adjuvant chemotherapy for luminal-type breast cancer was determined based on the risk for breast cancer recurrence and preference. Patients with HR-negative breast cancer were administered adjuvant chemotherapy. Patients with HER2-positive breast cancer were administered adjuvant trastuzumab concomitant with taxane-based chemotherapy and subsequent trastuzumab therapy for 1 year in total. Patients were followed in accordance with clinical guidelines that included clinical examination, annual mammography, and additional imaging tests in cases with signs of recurrence. Recurrence-free survival (RFS) was defined as the elapsed time from the date of surgery until the date of the first event (relapse or death from any cause) or of last follow-up.

### Evaluation of clinical node-negative and sentinel lymph node procedure

Clinical axillary nodal status was essentially evaluated by ultrasound sonography. Abnormal findings of axillary lymph nodes included the following: cortical thickness, abnormal morphologic characteristics, or loss of fatty hilum. Lymph nodes with abnormal findings were subsequently examined by fine needle aspiration cytology or core needle biopsy for the diagnosis of metastasis. Patients with negative findings from AUS and/or pathological evaluations by AUS-guided biopsy were diagnosed as having cN0. SLNB was performed to provide a final pathological diagnosis.

The SNLB procedure has been described in a previous report [13]. Briefly, SLNB is performed using both a dye colloid and radioisotopes. SLNB is performed at the same time along with the primary breast tumor resection. Sampled SLNs are evaluated for metastases using both the one-step nucleic acid amplification (OSNA) assay and histological evaluation. In the OSNA assay, OSNA− (CK19 mRNA < 2.5 × 10^2^ copies/μL) is diagnosed as negative, and OSNA+ (2.5 × 10^2^ to < 5.0 × 10^3^ copies/μL) and OSNA++ (≥ 5.0 × 10^3^ copies/μL) are diagnosed as positive for SLN metastases. In histological evaluation, no or isolated (< 0.2 mm) tumor cells is diagnosed as negative, and micrometastases (0.2–2 mm) or macrometastases (< 2 mm) is recognized as positive for SLN metastases. In OSNA assay, OSNA− is diagnosed as negative, and OSNA+ and OSNA++ are regarded as micrometastases and macrometastases, respectively.

During this study period, axillary lymph node dissections were essentially performed in cases with SLN-metastases. N stage was determined by the number of lymph node metastasis according to tumor, node, metastasis (TNM) staging classification for breast cancer.

### Statistical analysis

The association between clinicopathological factors and SLN status was assessed using the *χ*2 test. Kaplan–Meier survival curves and the log-rank test were used to determine the univariate significance of the variables. A Cox regression model was used to examine multiple covariates for survival.

Because the presence of SLN metastasis is thought to affect decisions regarding adjuvant chemotherapy or may be affected by tumor size or biological factors, a propensity score analysis was applied to control confounding factors. The propensity score matching included the following factors: age at surgery (< 55 vs. ≥ 55), T stage (T1 vs. T2), estrogen receptor status (positive vs. negative), progesterone receptor status (positive vs. negative), HER2 status (positive vs. negative), nuclear grade (1, 2 vs. 3), Ki67 index (< 14% vs. ≥ 14%), adjuvant chemotherapy (yes vs. no), and adjuvant radiation therapy (yes vs. no). Pairs of patients were identified using the propensity scores; one patient with SLN negative was randomly matched with a SLN positive, using the nearest matching neighbor within a caliper. The caliper coefficient was determined as 0.01. After adjustment with propensity score matching, differences in clinicopathology between groups were compared using the chi-square test.

A *p* value of < 0.05 was considered as statistically significant. Statistical analyses were done using JMP statistics software version 13.2.1 for Windows (SAS Institute, Inc.).

## Results

### Clinicopathological factors

Of the 582 patients with cT1–2N0 breast cancer, 471 patients (81%) were negative for SLN metastasis and 111 patients (19.1%) were positive for SLN metastasis, including 100 (17%) N1 and 10 (2%) N2 disease. Among 111 cN0SLN+ cases, the final pathological examination revealed 39 cases (6.7%) of micrometastasis and 72 cases (12.4%) of macrometastases. The median size of SLN metastasis was 3.0 mm (range 0.2–16 mm, mean 4.1 mm). Table 1 shows clinicopathological factors according to SLN status. Between the two groups, the probabilities of a T2 tumor (*p* < 0.0001) and the administration of adjuvant chemotherapy (*p* < 0.0001) were significantly higher in the SLN-positive group compared with the SLN-negative group. Otherwise, there was no significant difference in regard to age, ER, PgR, HER2, nuclear grade, Ki67 index, and adjuvant radiotherapy. Clinicopathological factors after propensity score matching are shown in Table 1.

**Table 1:** Clinicopathological factors according to the status of SLN in cN0 breast cancer before and after propensity score matching

Factor		All patients (*n* = 582)					Matched patients (*n* = 178)				
		cN0SN− (*n* = 471)		cN0SN+ (*n* = 111)			cN0SN− (*n* = 89)		cN0SN+ (*n* = 89)		
		*N*	%	*N*	%	*p* value	*N*	%	*N*	%	*p* value
Age	≥ 55	150	31.8	36	32.4	0.91	34	38.2	29	32.6	0.43
	< 55	321	68.2	75	67.5		55	61.8	60	67.4	
T factor	T1	354	75.2	57	51.4	< 0.0001	51	57.3	51	57.3	1
	T2	117	24.8	54	48.6		38	42.7	38	42.7	
ER	Positive	367	77.9	92	82.9	0.24	77	86.5	77	86.5	1
	Negative	104	22.1	19	17.1		12	13.5	12	13.5	
PgR	Positive	320	67.9	83	74.8	0.15	64	71.9	65	73	0.87
	Negative	151	22.1	28	25.2		25	28.1	24	27	
HER2	Negative	390	82.8	93	83.8	0.8	79	88.8	79	88.8	1
	Positive	81	17.2	18	16.2		10	11.2	10	11.2	
Nuclear grade	1, 2	345	73.3	84	75.7	0.6	69	67.5	69	67.5	1
	3	126	26.7	27	24.3		20	22.5	20	22.5	
Ki67	≥ 14%	170	36.1	31	27.9	0.11	22	24.7	22	24.7	1
	< 14%	293	63.9	77	72.1		67	75.3	67	75.3	
Adjuvant chemotherapy	Yes	153	32.5	66	59.5	< 0.0001	49	55.1	49	55.1	1
	No	318	67.5	45	40.5		40	44.9	40	44.9	
Adjuvant radiotherapy	Yes	308	65.4	69	62.2	0.52	56	62.9	57	64	0.88
	No	163	34.6	42	37.8		33	37.1	32	36	

*ER* estrogen receptor, *PgR* progesterone receptor, *HER2* human epidermal receptor 2

### Prognostic value of SLN metastasis in cT1–2N0 breast cancer

**Fig. 1:**
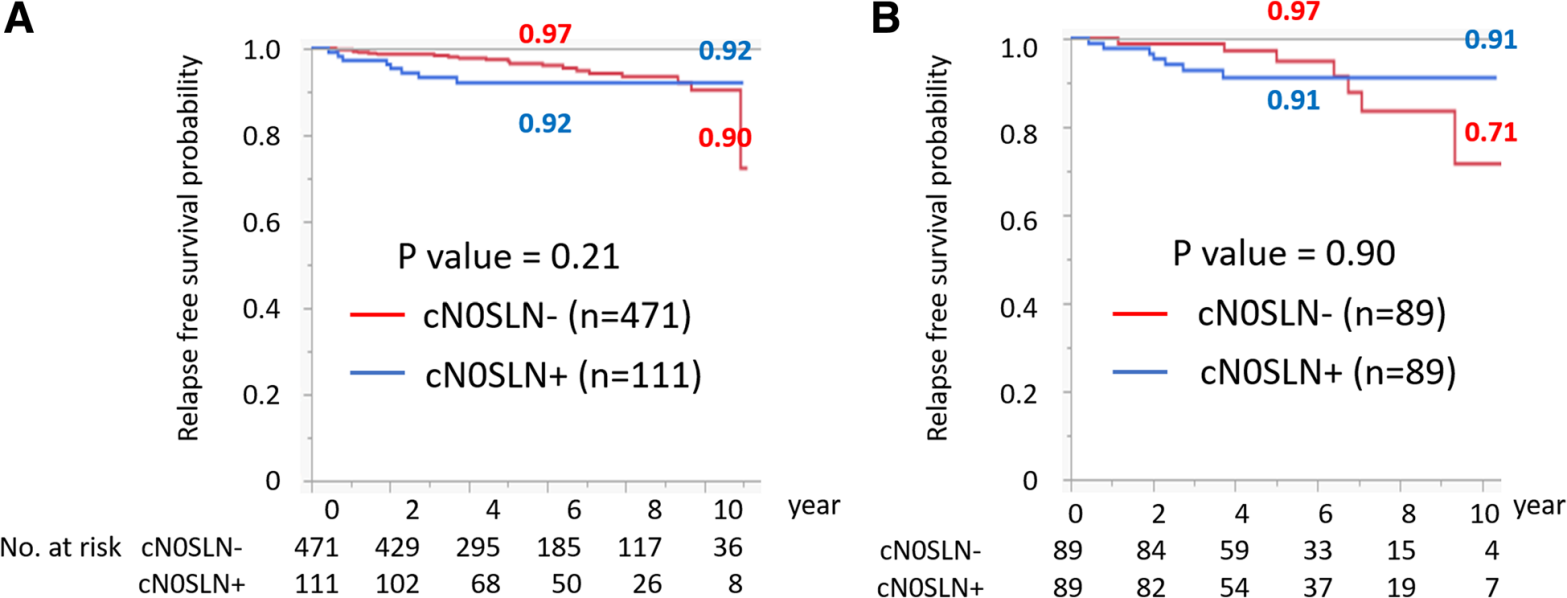
Prognostic value of SLN metastasis in cT1–2N0M0 breast cancer. Relapse-free survival **a** of all patients (*n* = 582) stratified by the presence of SLN metastasis and **b** of patients stratified by the presence of SLN metastasis among propensity score-matched patients (*n* = 178). *p* value was evaluated using the log-rank test. cN0 clinical node negative, SLN sentinel lymph node

**Fig. 2:**
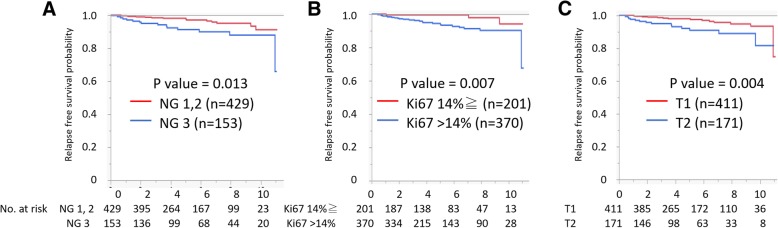
Prognostic value of Ki67 index and T stage in cT1–2N0M0 breast cancer. Relapse-free survival stratified by **a** nuclear grade, **b** Ki67 index, and **c** T stage. *p* value was evaluated using the log-rank test

Within the median follow-up period from definitive surgery for primary breast cancer of 4.9 years, 27 patients (4.6%) had breast cancer recurrences. In univariate analysis with a log-rank test, the SLN status was not a significant prognostic factor (*p* = 0.21) (Fig. 1a). The 5-year and 10-year RFS were 93.0%, 96.5%, and 93.0%, 90.4% in the SLN-positive and SLN-negative group, respectively. In regard to the size of metastases, the 5-year and 10-year RFS were 94.0%, 91.0%, and 94.0%, 91.0% in SLN micrometastases and SLN macrometastases, respectively (log-rank test, *p* = 0.57) (Additional file 1: Figure S1). In the propensity score matching cohort (*n* = 178), there was no significant difference in RFS between a SLN-positive and SLN-negative group (*p* = 0.90) (Fig. 1b). On the other hand, nuclear grade 3 (*p* = 0.013), a higher Ki67 index (*p* = 0.007), and T2 (*p* = 0.004) were significantly associated with worse RFS in a log-rank test (Fig. 2). There was a tendency for a worse prognosis in ER-negative (*p* = 0.11) breast cancer. Cox regression analysis showed that a continuous value of Ki67 expression was a significant prognostic factor (HR 1.03; 95% CI, 1.01–1.05, *p* = 0.017). A higher Ki67 index (> 14%) was a marginal significant poor prognostic factor for RFS (HR 3.16, 95% confidential interval 0.99–14.02, *p* = 0.051). On the other hand, the presence of SLN metastasis was not a significant prognostic factor (*p* = 0.44). In exploratory analysis, the prognoses of patients with cT1–2cN+M0 breast cancer were compared with patients with cT1–2cN0M0 breast cancer, and there was a significant difference in RFS between these groups. The 5-year and 10-year RFS were 92.0%, 70.0%, and 92.0%, 68.0% in the cN0SLN-positive and cN-positive group, respectively (*p* < 0.0001, log-rank test) (Additional file 2: Figure S2). cN positive was still a significant poor prognostic factor in Cox regression analysis (HR 4.4, 95% CI 1.8–11.8, *p* = 0.0006) (Table 2).

**Table 2:** Multivariate analyses of predictors for distant disease-free survival and overall survival (*n* = 582)

Factor	HR	(95% CI)	*p* value
Age (≤ 55 vs. > 55)	1.3	(0.55–3.33)	0.56
T factor (T2 vs. T1)	1.56	(0.66–3.64)	0.3
SLN factor (SLN+ vs. SLN−)	1.42	(0.56–3.36)	0.44
ER (positive vs. negative)	0.81	(0.26–2.7)	0.73
PgR (positive vs. negative)	1.21	(0.42–3.82)	0.73
HER2 (positive vs. negative)	0.64	(0.20–1.69)	0.38
Nuclear grade (3 vs. 1, 2)	1.39	(0.53–3.64)	0.51
Ki67			
Ki67 (< 14% vs. ≥ 14%)	3.16	(0.99–14.02)	0.051
Ki67 (continuous value)	1.03	(1.01–1.05)	0.017
Adjuvant chemotherapy (yes vs. no)	1.2	(0.48–3.09)	0.69
Adjuvant radiotherapy (yes vs. no)	0.71	(0.31–1.69)	0.29

*ER* estrogen receptor, *PgR* progesterone receptor, *HER2* human epidermal receptor 2, *SLN* sentinel lymph node

## Discussion

In this study, we showed that the presence of SLN metastases has a minimal impact on prognosis in patients with cT1–2N0M0 breast cancer in the modern medical era. The 5-year and 10-year RFS were 93.0%, 96.5%, and 93.0%, 90.4% in a SLN-positive and SLN-negative group, respectively, and there was no significant difference in RFS between these groups (*p* = 0.2, log-rank test). After propensity score matching between the cN0SLN+ group and the cN0SLN− group, there was still no significant difference in RFS. On the other hand, a proliferation marker is a better factor for poor prognosis than the presence of SLN metastases in this population.

The presence of axillary lymph node metastasis has been regarded as an important prognostic factor in patients with non-metastatic breast cancer [14, 15]; however, the prognostic value is considered to be influenced by tumor burden in involved lymph nodes in patients who underwent SLN biopsy for cN0 breast cancer. Isolated tumor cell (ITC) (not greater than 0.2 mm) in SLN is regarded as pathological node negative (pN0) because the presence of ITC does not confer a deteriorated prognosis. The clinical significance of micrometastases (> 0.2 mm but < 2.0 mm) in breast cancer remains controversial; prospective observational analyses by Andersson et al. showed poor prognostic significance for micrometastases in SLN [16, 17]; however, data from a NSABP B32 trial, ACOSOG Z0010 trial, and MD Anderson Cancer Center showed minimal impact from micrometastases on survival outcome in patients who underwent SLN biopsy [18, 19]. The presence of macrometastases in SLNs (> 2.0 mm) is conventionally regarded as an established poor prognostic factor; however, the prognostic value of macrometastases in SLN may be decreasing in the modern medical era. A previous report of 647 patients with cT1–2N0 breast cancer by Tucker et al., in which cN0 was diagnosed by AUS, patients with false-negative results of AUS had an equivalent RFS to patients with a pathologic node-negative disease [5]. Our study also evaluated cN0 by AUS and/or AUS-guided biopsy and showed no significant difference in RFS between a SLN-positive and SLN-negative group in patients with cT1–2N0M0 breast cancer. In this study, because the proportion of adjuvant chemotherapy is significantly affected by the presence of SLN metastases, propensity score analysis was applied to control confounding factors. After the propensity score matching, there was still no significant difference in RFS between the cN0SLN+ and cN0SLN− group. On the other hand, cN+ is still a significant prognostic factor in cT1–2 breast cancer. The prognosis of patients with cT1–2 and cN+ breast cancer was compared with those with cT1–2cN0 and SLN+ breast cancer, and the 5-year and 10-year RFS were 92.0%, 70.0%, and 92.0%, 68.0% in the cN0SLN+ and cN+SLN group, respectively. The previous report by Tucker et al. also showed that the RFS for patients with cN+ disease was significantly worse than for patients with cN0 and SLN+ disease. These findings suggest that clinically detectable lymph node metastasis has a clinically meaningful tumor burden, which has a significant survival impact for patients with breast cancer.

The decreased impact of SLN metastasis on breast cancer survival can be explained by advances in axillary imaging evaluation and adjuvant systemic therapy. First, AUS-based axillary evaluation prior to axillary surgery can exclude a significant tumor burden in lymph nodes. In this study, cN0 diagnosed by AUS or AUS-guided needle biopsy showed a false-negative rate of 19.1%, including 6.7% of micrometastasis and 12.4% of macrometastases. This result is similar with the previous report by Mittendorf et al. [19]; the false-negative rate was 22.6%, including 8.8% of micrometastasis and 13.8% of macrometastases. In contrast, a previous report by Andersson et al. showed a higher probability of false negatives (25.8%) and macrometastases (22.2%), in which the presence of SLN metastases was evaluated as a significant factor for poor prognosis. The tumor burden of false-negative lymph nodes may affect the prognostic significance of SLN metastases. In regard to the accuracy of AUS for detection of axillary disease, recent studies have shown consistent and sufficient results. Cyr et al. reported the ability of AUS to exclude clinically significant metastases in the axillary node, and the NPV of AUS for identification of macrometastases was 96.9% in cT1–2N0 breast cancer [4]. Nakamura et al. reported that a proportion of patients with a significant number of lymph node metastases (≥ 3) was 16% and 5% in a cN0-FNA group and cN0-CNB group, respectively [7]. These findings show that preoperative axillary evaluation by AUS can exclude clinically significant metastases of axillary lymph nodes in cT1–2N0M0 breast cancer. Second, presences of lymph node metastases seem to have less impact on survival in cN0 breast cancer compared with biologic factors. In regard to micrometastases, retrospective analyses of phase III trial and large observational study showed no significant impact on prognosis of occult metastases in cN0 breast cancer [18, 19]. In these analyses, endocrine responsiveness and grade were more important prognostic factors compared with micrometastases. Our study and a retrospective study by Tucker et al. [5] also showed equivalent RFS of patients with cN0 and pathologically node-positive disease to those with cN0 and pN0 disease; however, two thirds of node-positive patients had macrometastases in these studies. In these studies, cN0 was essentially diagnosed by AUS. In addition, the proliferation marker Ki67 index was a significant poor prognostic factor for RFS in our study. Third, during the period of this study, guidelines for adjuvant therapy have changed to emphasize the responsiveness [9]. In luminal subtype, adjuvant chemotherapy was conventionally determined by risk category defined by nodal status, T stage, grade, vascular invasion, and age [20]; however, recent adjuvant chemotherapy recommendation is based on the endocrine responsiveness. In luminal A-like subtype, adjuvant chemotherapy is considered in a case with four or more node involvement. In luminal B-like subtype, adjuvant chemotherapy is recommended irrespective of nodal status [21]. In HER2 subtype, adjuvant chemotherapy with HER2-targeting therapy was not indicated in a patient with a primary tumor < 1 cm of size and with no axillary node involvement, and recent guideline expanded the indication of adjuvant therapy to T1bN0 HER2-positive disease considering the substantial recurrence risk [22]. In TNBC, adjuvant chemotherapy is essential in both node-positive and node-negative disease. These findings suggest that tumor biology is more important for deciding adjuvant therapy and predicting recurrence than SLN metastases in patients with cT1–2N0M0 breast cancer. Finally, the therapeutic value of axillary surgery is denied in patients with clinical T1–T2N0 (cT1–2N0) breast cancer. In cN0 breast cancer, an omission of axillary lymph node dissection (ALND) resulted in an increment of the rate of axillary failure; however, this local failure did not lead to an increment of distant metastasis or a survival disadvantage [23]. In cT1–2N0SLN+ breast cancer treated with appropriate adjuvant systemic therapy and radiotherapy, ALND failed to show an improvement of local control nor long-term prognosis in spite of the possibility of residual disease in non-SLN [2, 3]. Thus, omission of axillary surgery itself does not result in deteriorated prognosis in patients with cT1–2N0M0.

There are ongoing randomized clinical trials comparing SLN biopsy to no axillary surgery in patients with cT1–2N0M0 breast cancer, in which cN0 is evaluated by AUS and/or ultrasonography-guided biopsy [4, 24]. Because SLN biopsy is associated with surgical complications including pain, paresthesias, seroma, lymphedema, and delayed wound healing, SLN biopsy should be spared if these randomized studies show the effectiveness and safety of omission of SLN biopsy in cT1–2 N0 breast cancer. Considering the possibility of omission of axillary surgery, clinical diagnosis of cN0 disease becomes crucially important. Although several innovative techniques, such as contra-enhanced AUS, MRI, or PET-CT, are utilized for detection of lymph node metastasis, morphological evaluation by AUS is still the standard procedure for preoperative axillary staging. Although the ability of AUS for detection of lymph node metastasis is dependent on an individual clinician’s skill, the accuracy of AUS for detection of lymph node metastasis seems to be similar across studies. Indeed, the negative predictive value of 81% for detection of lymph node involvement in our study is consistent with previous reports. Considering this consistency and universality, AUS is the preferable procedure for exclusion of clinical significant lymph node metastasis.

Our study has several limitations. One limitation was the relatively short follow-up period (median, 4.9 years). However, we think that this follow-up period is enough to show the minimal prognostic impact of SLN metastasis in patients with cT1–2 and cN0 breast cancer. In luminal subtype, the annual recurrence rate is consistent up to 10 years and early recurrence is a certain surrogate marker of late recurrence. In non-luminal subtype, most recurrence occurs within 5 years and early recurrence is the primary endpoint for survival. Although the evaluation of late recurrence may confer additional survival information, the prognostic value of SLN metastasis in this study will not be altered. Second, this retrospective analysis from a single institution could have biases, and a multiple-institutional prospective study is needed to confirm our results. Finally, there was a different proportion of subtype in recurrent breast cancer between SLN+ and SLN− groups in this study. In SLN+ group, 4 out of 8 recurrent cases were non-luminal subtype, which contributed the increment of early recurrence. On the other hand, 16 out of 20 recurrent cases were luminal subtype, which contributed the increments of late recurrence. This uneven distribution of subtype in recurrent cases leaded the different relapse-free survival curves before 5 years and at 10 years in SLN-positive and SLN-negative groups.

In conclusion, our study showed that the presence of SLN metastasis has a minimal impact on prognosis in patients with cT1–2N0M0 breast cancer. A proliferation marker is a better factor for poor prognosis than occult nodal metastases. SLN biopsy may be spared if prospective randomized studies show the effectiveness and safety of omission of SLN biopsy in cT1–2N0M0 breast cancer.

## Additional files

Additional file 1: **Figure S1.** Relapse-free survival stratified by the size of SLN metastasis. *p* value was evaluated using the log-rank test. Abbreviations: cN0: clinical node negative, SLN: sentinel lymph node. (TIF 163 kb)

Additional file 2: **Figure S2**. Relapse-free survival stratified by cN status and SLN status. *p* value was evaluated using the log-rank test. Abbreviations: cN0: clinical node negative, SLN: sentinel lymph node. (TIF 223 kb)
