# Supplementary figures and images for: Minimal prognostic significance of sentinel lymph node metastasis in patients with cT1–2 and cN0 breast cancer
Source: World J Surg Oncol. 2019 Feb 23;17:41. doi: 10.1186/s12957-019-1585-9 (PMC6387738; doi:10.1186/s12957-019-1585-9)

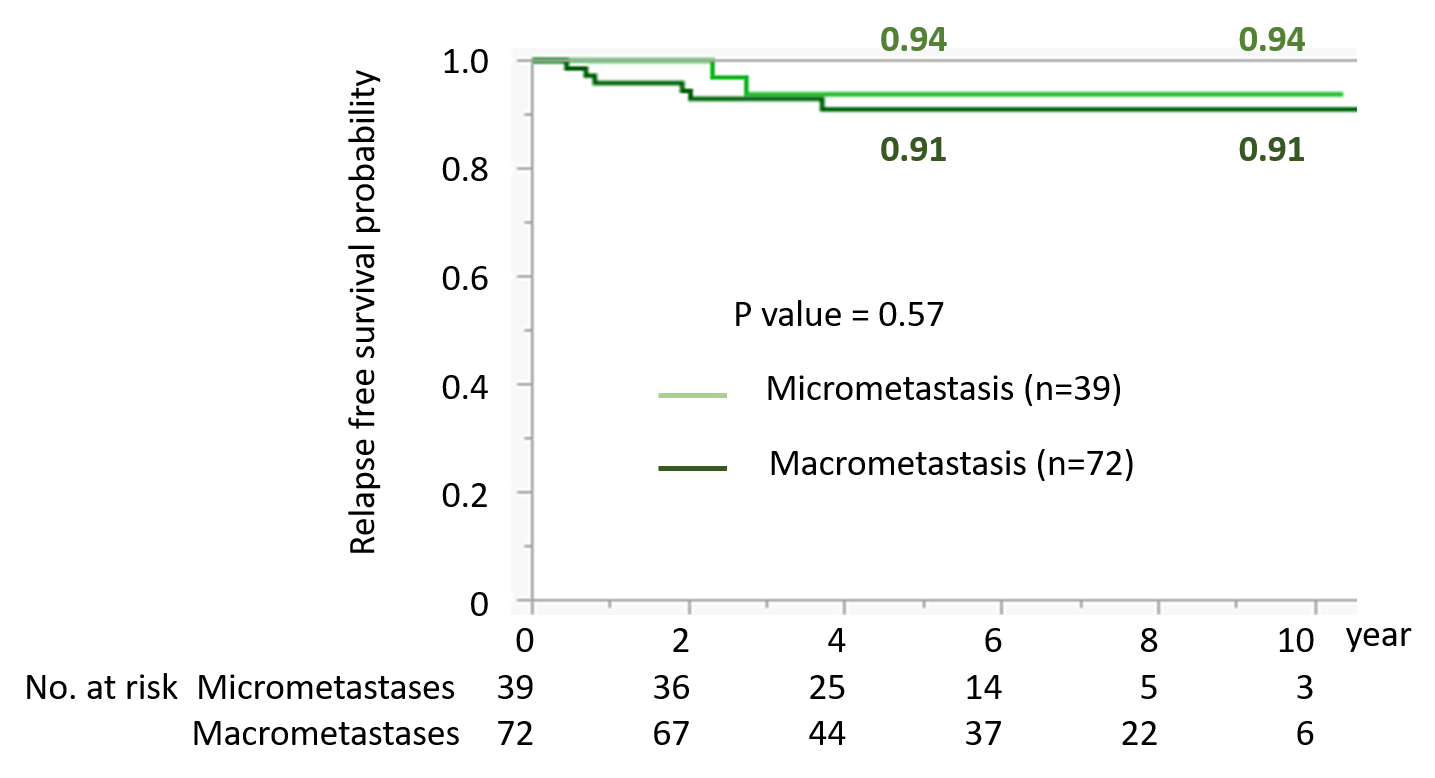

Supplement: Supplementary file 1 — Figure S1. Relapse-free survival stratified by the size of SLN metastasis. p value was evaluated using the log-rank test. Abbreviations: cN0: clinical node negative, SLN: sentinel lymph node. (TIF 163 kb) [file 12957_2019_1585_MOESM1_ESM.tif]

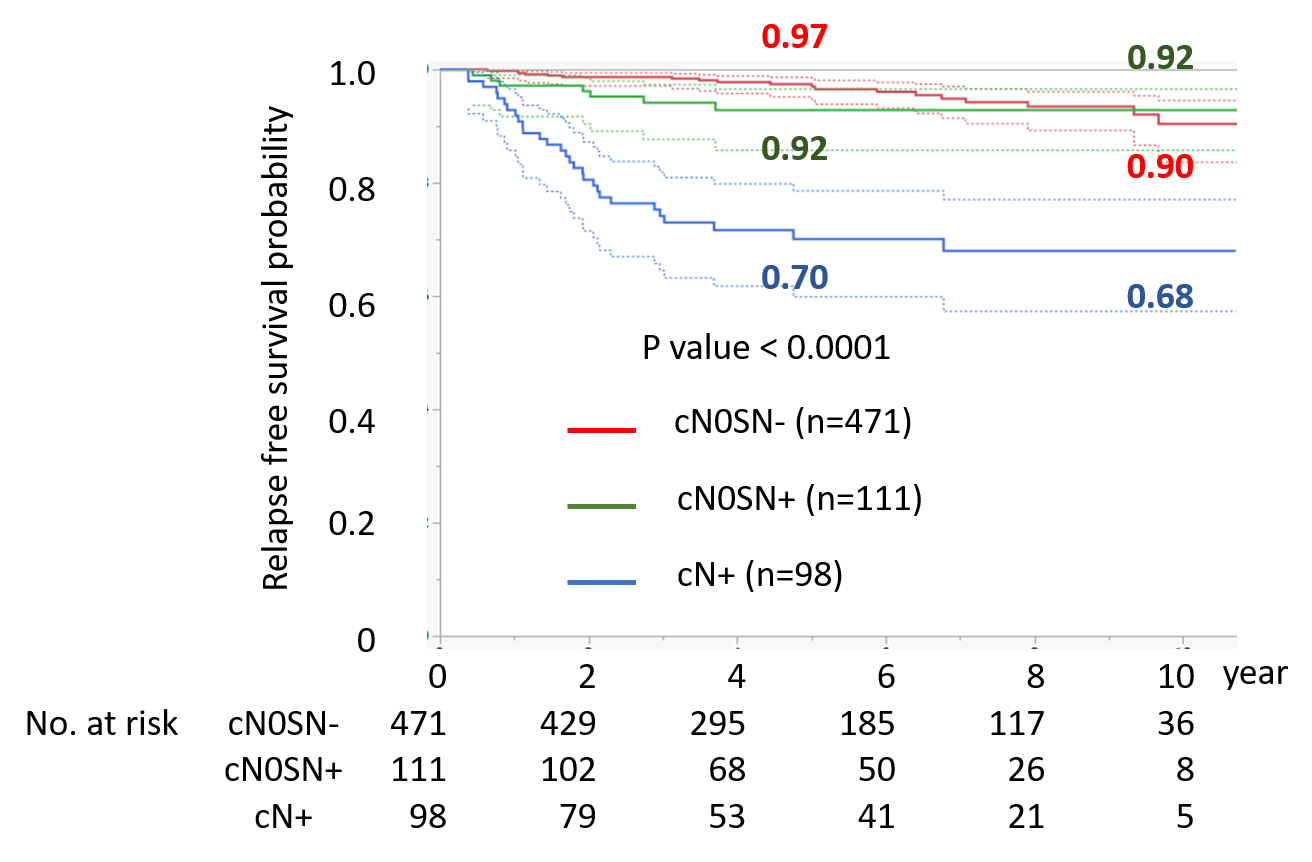

Supplement: Supplementary file 2 — Figure S2. Relapse-free survival stratified by cN status and SLN status. p value was evaluated using the log-rank test. Abbreviations: cN0: clinical node negative, SLN: sentinel lymph node. (TIF 223 kb) [file 12957_2019_1585_MOESM2_ESM.tif]
